# REDUCE trial: the effects of pe*r*in*e*ural *d*examethasone on scalp nerve blocks for relief of postcraniotomy pain—a st*u*dy proto*c*ol for a randomiz*e*d controlled trial

**DOI:** 10.1186/s13063-021-05747-y

**Published:** 2021-11-04

**Authors:** Chunmei Zhao, Zipu Jia, Niti Shrestha, Fang Luo

**Affiliations:** 1 Department of Pain Management, Beijing Tiantan Hospital, Capital Medical University, Beijing, 100070 China; 2 Department of Day Surgery, Beijing Tiantan Hospital, Capital Medical University, Beijing, China

**Keywords:** Postoperative pain, Craniotomy, Dexamethasone, Scalp nerve blocks, Randomized controlled trial

## Abstract

**Background:**

Pain is common in the first 2 days after major craniotomy. Inadequate analgesia may lead to an increased risk of postoperative complications. Most pain following craniotomy arises from the pericranial muscles and soft tissues of the scalp. Scalp nerve blocks with local anesthesia seem to provide effective, safe, however, transient postoperative analgesia which does not seem to meet the requirements of craniotomy. Currently, peripheral dexamethasone has been observed to significantly prolong the duration of analgesia of nerve blocks (e.g., saphenous nerve block, adductor canal block, thoracic paravertebral block, brachial plexus nerve block). On the contrary, a study reported that perineural dexamethasone did not appear to prolong the analgesic time after supratentorial craniotomy. However, all patients in this study were given 24 mg of oral or intravenous dexamethasone regularly for at least 7 days during the perioperative period, which possibly masked the role of single local low doses of perineural dexamethasone. Therefore, the analgesic effect of single dexamethasone for scalp nerve blocks without the background of perioperative glucocorticoid deserves further clarification.

**Methods:**

The REDUCE trial is a prospective, single-center, parallel-group randomized controlled trial involving a total of 156 adults scheduled for elective craniotomy with general anesthesia. Patients will be randomly divided among two groups: the control group (*n* = 78) will receive scalp nerve blocks with 0.5% bupivacaine, plus normal saline with epinephrine at 1:200,000; the DEX_4mg_ group (*n* = 78) will receive scalp nerve blocks with 0.5% bupivacaine, plus 4 mg dexamethasone with epinephrine at 1:200,000. The primary outcome will be the duration of analgesia, defined as the time between the performance of the block and the first analgesic request.

**Discussion:**

The REDUCE trial aims to further assess the analgesic effect of single dexamethasone as an adjuvant to scalp nerve blocks for relief of postcraniotomy pain without the background of perioperative glucocorticoid.

**Trial registration:**

ClinicalTrials.govNCT04648358. Registered on November 30, 2020.

**Supplementary Information:**

The online version contains supplementary material available at 10.1186/s13063-021-05747-y.

## Administrative information

Note: the numbers in curly brackets in this protocol refer to SPIRIT checklist item numbers. The order of the items has been modified to group similar items (see http://www.equator-network.org/reporting-guidelines/spirit-2013-statement-defining-standard-protocol-items-for-clinical-trials/).

**Table:** 

Title {1}	REDUCE trial: The effects of Perineural Dexamethasone on Scalp Nerve Blocks for Relief of Postcraniotomy Pain: a study protocol for a randomized controlled trial
Trial registration {2a and 2b}.	Clinicaltrials.gov, NCT04648358. Registered on November 30, 2020.
Protocol version {3}	2018/07/23 Protocol Version 3.2
Funding {4}	This research was supported by the Capital’s Funds for Health Improvement and Research (No. 2020-2-2046).
Author details {5a}	Chunmei Zhao*, Department of Pain Management, Beijing Tiantan Hospital, Capital Medical University, Beijing, China. Zipu Jia*, Department of Day Surgery, Beijing Tiantan Hospital, Capital Medical University, Beijing, China. Niti Shrestha*, Department of Pain Management, Beijing Tiantan Hospital, Capital Medical University, Beijing, China. Fang Luo, Department of Pain Management, Beijing Tiantan Hospital, Capital Medical University, Beijing, China. E-mail: 13611326978@163.com
Name and contact information for the trial sponsor {5b}	Capital’s Funds for Health Improvement and Research. Contact information:0086-010-59976664
Role of sponsor {5c}	The funder is not involved in subject recruitment, intervention, data collection, data analysis or preparation of the manuscript.

## Introduction

### Background and rationale {6a}

Pain is often considered an inevitable outcome of surgery. Postcraniotomy patients have frequently been assumed to experience modest pain among all surgeries. However, current evidence suggests that pain after neurosurgical procedures is more severe than expected [1, 2]. A prospective study of 187 patients demonstrated that pain was common in the first 2 days after major craniotomy, with approximately 70% of patients reporting moderate to severe pain during the 1st postoperative day and 48% of patients complaining of pain scores greater than or equal to 4 on the 2nd postoperative day [2]. Sympathetically mediated hypertension caused by inadequate analgesia may lead to an increased risk of postoperative complications, such as arterial hypertension, intracranial hemorrhage, prolonged hospital stays, and mortality [3]. In addition, undertreated acute postoperative pain may predict the development of chronic pain [4–6]. Therefore, optimal pain management is of great importance for improving postoperative recovery and patient comfort [7].

Opioids are commonly used for postoperative analgesia, but the use of opioids for neurosurgical procedures is limited by potential side effects such as sedation, miosis, nausea, vomiting, and respiratory depression [8]. Multimodal analgesic techniques, such as combining an opioid with supplemental analgesics (including nonsteroidal anti-inflammatory drugs (NSAIDs), scalp infiltration, and scalp nerve blocks), typically result in improved analgesia with concurrent reductions in the incidence of systemic opioid-related adverse effects [8, 9]. The neural tissue of the brain does not contain any pain receptors. Most pain following craniotomy arises from the pericranial muscles and soft tissues of the scalp [10]. Therefore, scalp nerve blocks, which suspend the function of the sensory nerve fibers in the superficial and deep soft tissue layers, can reduce the consumption of analgesic drugs and may enhance early postoperative recovery.

Scalp nerve block is a widespread technique that has been utilized in anesthetic and neurosurgical practice for several decades. Scalp nerve blocks with local anesthetics seem to enable effective and safe anesthetic management and are also critical for awake craniotomy procedures [11–13]. In neurosurgery, scalp nerve blocks can be performed by directly blocking several different nerves such as the supraorbital and supratrochlear nerves, the auriculotemporal nerves, the zygomaticotemporal nerves and the greater, lesser, and third occipital nerves that provide sensory innervation of the scalp in neurosurgery [14]. Lee et al. suggested that 0.25% bupivacaine scalp nerve blocks could effectively attenuate the hemodynamic response to skin incision and dural opening in patients undergoing craniotomy with general anesthesia [15]. There was also evidence that the pain intensity was reduced after scalp nerve blocks with local anesthetics in the first few hours after craniotomy [16–19]. Even with adrenaline used as an additive agent, scalp nerve blocks using 0.5% or 0.75% bupivacaine with adrenaline improved postoperative analgesia for a maximum of only 6 h after craniotomy [20, 21]. On the contrary, Rigamonti et al. revealed that scalp nerve blocks with 0.5% bupivacaine with adrenaline did not decrease the severity of postoperative pain for patients undergoing supratentorial craniotomy [22]. Therefore, to meet the analgesic requirements of craniotomy, improving the quality and prolonging the duration of scalp nerve blocks is of great significance. Continuous perineural catheter is a common technique to prolong the effect of local anesthetics. However, there are potential risks with these catheters themselves (e.g., dislocation, prolonged motor block, infection) [23–25]. Adjuvants have been used to prolong the duration of postoperative analgesia to avoid these defects. Among the different adjuvants, the potent long-acting glucocorticoid agonist dexamethasone is one of the most acceptable adjuvants.

In 2003, Shrestha et al. firstly reported that peripheral dexamethasone significantly prolonged the duration of analgesia of brachial plexus block [26]. Subsequently, the addition of dexamethasone (1~8 mg) was observed to prolong the median duration of analgesia by 27~39% in various nerve blocks (e.g., saphenous nerve block, adductor canal block, thoracic paravertebral block, brachial plexus nerve block) without any unwanted effects [27–36]. Among them, a meta-analysis of optimal dose of perineural dexamethasone suggested that 4 mg of perineural dexamethasone represents an analgesic ceiling dose and higher doses failed to provide additional analgesic duration [34]. The possible mechanisms of perineural dexamethasone prolonging the analgesic time of the peripheral nerve block are by reducing local anesthetics absorption by inducing a degree of vasoconstriction [37] and decreasing activity of C fibers by inhibiting potassium channels [38]. Moreover, there is no evidence of significant neurotoxicity of perineural administration of dexamethasone [39]. On the contrary, in a pioneering study, Jose et al. applied 8 mg dexamethasone as an adjuvant to 0.2% ropivacaine for scalp nerve blocks and reported that perineural dexamethasone did not appear to prolong the analgesic time after supratentorial craniotomy. However, all patients were given 24 mg of oral or intravenous dexamethasone regularly at least 7 days during the perioperative period. Such persistent high doses of systemic dexamethasone possibly masked the role of single local low doses of perineural dexamethasone. The analgesic effect of single dexamethasone for scalp nerve blocks without the background of perioperative glucocorticoid deserves further clarification.

We postulate that without the background of perioperative glucocorticoid, the use of single dexamethasone as an adjuvant to scalp nerve blocks can prolong the analgesic duration, reduce opioid consumption and improve the quality of scalp nerve blocks compared with bupivacaine alone after supratentorial craniotomy. The primary outcome will be the duration of analgesia, defined as the time between the performance of the block and the first analgesic request.

### Objectives {7}

We aim to assess the effectiveness of scalp nerve blocks with local anesthetics plus dexamethasone for the relief of postcraniotomy pain without the background of perioperative glucocorticoid.

### Trial design {8}

**Fig. 1:**
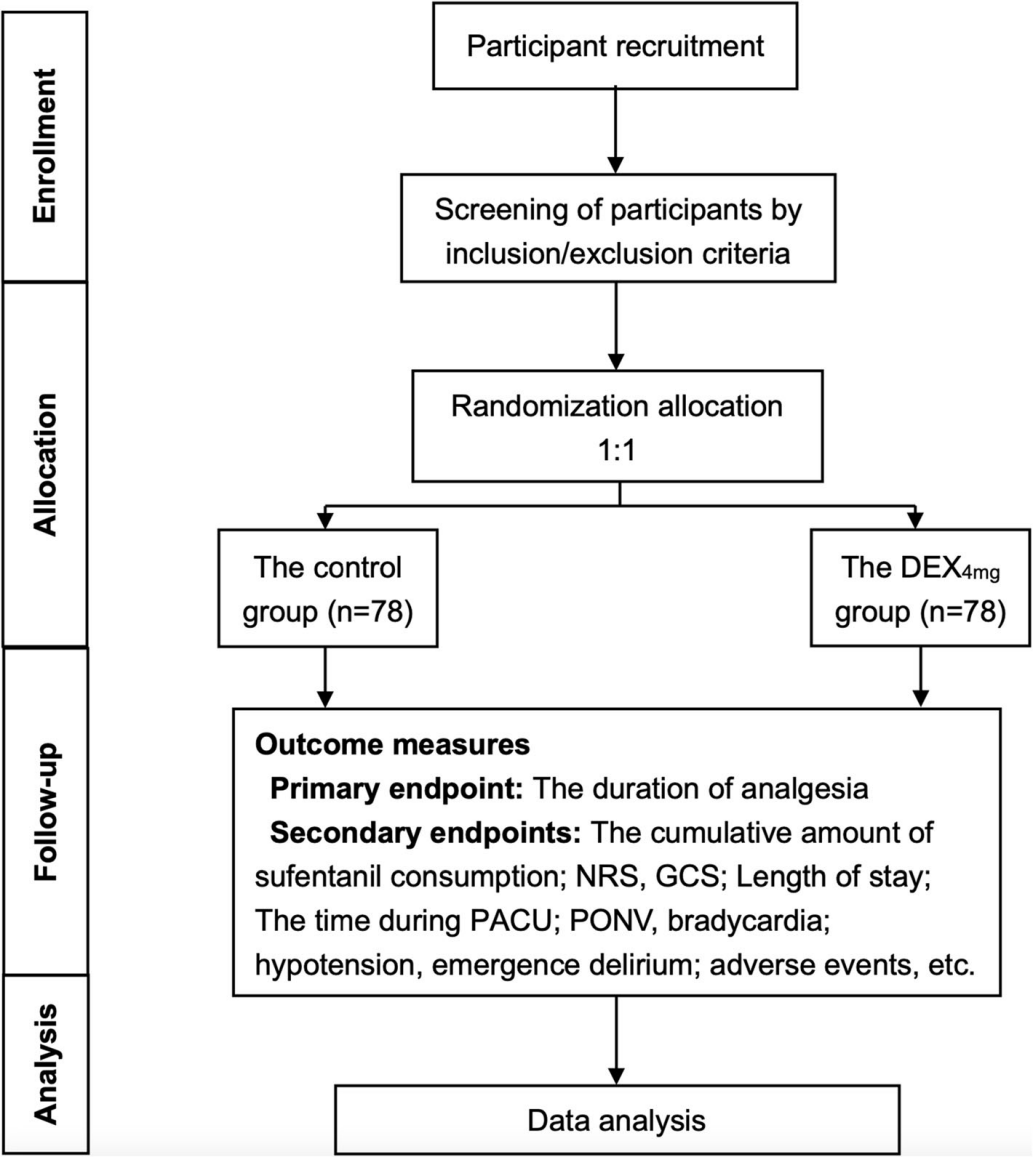
Flow chart of the study procedure. DEX, dexamethasone; NRS, Numeric Rating Scale; GCS, Glasgow Coma Scale score; PACU, postoperative care unit; PONV, postoperative nausea and vomiting

This study is a prospective, single-center, parallel-group randomized controlled, superiority trial. The planned study has been registered at ClinicalTrials.gov under the identifier NCT04648358. See Fig. 1 for the flow chart. Additional File 1 shows the Standard Protocol Items: Recommendations for Interventional Trials (SPIRIT) checklist for study protocols.

## Methods: Participants, interventions, and outcomes

### Study setting {9}

A high-volume center with broad expertise in neurosurgery will be involved in enrollment (Beijing Tiantan Hospital, Capital Medical University). Recruitment of patients for the REDUCE trial started in December 2020, and the trial is expected to complete in 2022.

### Eligibility criteria {10}

#### Inclusion criteria

Patients scheduled for elective supratentorial craniotomy under general anesthesia will be consecutively screened for eligibility based on the following criteria: age 18 to 64 years; an American Society of Anesthesiologists (ASA) physical status of I, II or III; and a preoperative Glasgow Coma Scale (GCS) score of 15/15. Each participant must be able to understand the nature and potential individual consequences of the clinical trial.

#### Exclusion criteria

1. History of chronic headache or chronic pain syndrome of any cause, psychiatric disorders or uncontrolled epilepsy;
2. Inability to understand or use the pain scales before surgery;
3. Excessive alcohol or drug abuse, chronic opioid use (more than 2 weeks or 3 days per week for more than 1 month), use of drugs with confirmed or suspected sedative or analgesic effects, or use of any painkiller within 24 h before surgery;
4. Request of oral/intravenous glucocorticoid to decrease cerebral edema within 1 week before surgery;
5. Pregnancy or breastfeeding;
6. Extreme body mass index (BMI) (< 15 or > 35);
7. Participation in another interventional trial that interferes with the intervention or outcome of this trial;
8. Refusal or inability of the patient and/or legal guardian to provide informed consent;
9. Coagulopathy;
10. Infection around the puncture site; and
11. History of allergies to any of the study drugs.

#### Subject withdrawal criteria

Subjects may withdraw from the study under the following circumstances: subjects cannot complete this trial, or they request, for various reasons, that they not be subjected to the intervention; oral/intravenous glucocorticoid is required to decrease cerebral edema intraoperatively or postoperatively; the planned craniotomy is not performed; or adverse events (AEs) force subjects to withdraw from the trial. Subjects who withdraw will be included in the final report of the REDUCE trial to ensure full transparency.

### Who will take informed consent {26a}

Patients who want to take part in the study can communicate with the study coordinator to learn more about the study. A study coordinator will explain the rationale and methodology of the study face to face to the patient or legal representative. The patient or legal representative has the option to ask questions and the opportunity to consider whether to participate in this trial. If the patient is willing, consent will be obtained for the REDUCE trial. Then, written informed consent documents will be securely locked in one place.

### Additional consent provisions for collection and use of participant data and biological specimens {26b}

Permission for the research team to share relevant data will be obtained from participants. Participants will also be asked whether they agree to use their data if they withdraw from this trial. In addition, the participants will be provided opportunities to ask any questions about the use of analgesia pump use and data collection. The REDUCE trial will not involve the collection of biological specimens.

## Interventions

### Explanation for the choice of comparators {6b}

A meta-analysis pointed out that perineural administration of dexamethasone as an adjuvant to bupivacaine, but not ropivacaine, could slightly prolong the duration of analgesia [35]. Due to the elevated pH of dexamethasone and the incompatibility of ropivacaine with alkaline solutions, in vitro study observed rapid crystallization with a mixture of dexamethasone and ropivacaine. Therefore, the combination of dexamethasone and ropivacaine should be used with caution. The combination with dexamethasone and bupivacaine did not observe such phenomenon and will therefore be used in the REDUCE trial.

### Interventions description {11a}

#### Intervention and control groups

After the initiation of the study, eligible participants will be consecutively screened for trial inclusion. A total of 156 participants will be randomly assigned to one of the two groups using a computer-generated list.

The control group will receive scalp nerve blocks with 0.5% bupivacaine 21 ml with epinephrine at 1:200,000, plus normal saline 1 ml.

The DEX_4mg_ group will receive scalp nerve blocks with 0.5% bupivacaine 21 ml with epinephrine at 1:200,000, plus 4 mg dexamethasone (1 ml).

An independent researcher will prepare the study solution in a separate operating room. After opening the envelope containing the treatment allocation, the study solutions will be prepared in 50-ml syringes for scalp nerve blocks with 23-gauge needles and will be numbered by an independent researcher. After induction, the assigned solutions will be used for nerve blocks injections by the anesthesiologist, who will be blinded to the group allocations.

#### Anesthesia protocol

All participants will be informed about the REDUCE trial during a pretreatment visit. Each participant will be equally randomized to one of the groups using a closed envelope technique. After the patient arrives in the operating room, standard monitoring, such as a 5-lead electrocardiogram, heart rate (HR), pulse oximetry, and noninvasive arterial blood pressure (BP) will be recorded. Induction will be carried out with 2 to 3 mg/kg propofol and 0.3 to 0.5 μg/kg sufentanil. Neuromuscular blockade will be performed with 0.6 mg/kg I.V. rocuronium to facilitate tracheal intubation.

After induction, anesthesia will be maintained with 4 to 8 mg/kg/h propofol and 0.1 to 0.3 μg/kg/min remifentanil at the discretion of the attending anesthesiologist. Remifentanil will be adjusted according to the degree of surgical stimulation. Additional boluses of remifentanil (0.5 μg/kg/bolus) will be administered to decrease any significant response to surgical stimulation. Meanwhile, the infusion rate of remifentanil will be increased by 0.05 μg/kg/min. No additional sufentanil will be used intraoperatively. We will use mechanical ventilation through an endotracheal tube with a mixture of oxygen in air (fraction of inspired oxygen (FiO2) 0.5 to 0.6) with end-tidal carbon dioxide between 35 and 45 mmHg. The intraoperative HR and BP will be maintained within 20% of the baseline levels. An increase or decrease in the mean arterial pressure (MAP) from the baseline value by more than 20% will be considered clinically significant and will be treated with nicardipine or dopamine. Nicardipine or dopamine will be administered with intermittent boluses and with continuous infusion if necessary. The number of patients who receive nicardipine or dopamine will be recorded. The intraoperative HR and BP will be recorded before anesthetic induction, after anesthetic induction, 5 min after intubation, and at the insertion of cranial pins, skin incision, skull drilling, dura mater opening, and skin closure. Similarly, any significant elevation or reduction in HR and BP will be noted throughout the operation. The dosages of all drugs and intraoperative physical parameters will be closely monitored and recorded by the investigator.

After surgery, neostigmine (0.05 mg/kg) and atropine (0.02 mg/kg) will be administered to reverse residual muscle relaxation. Ondansetron (8 mg) as an antiemetic prophylaxis will be intravenously administered. Extubation will be performed when the standard extubation criteria, such as eye opening, adequate spontaneous respiration, and purposeful movements, have been met.

#### Scalp nerve blocks

The anesthesiologist will perform scalp nerve blocks based on the group allocation 10 mins before the incision. Scalp nerve blocks will be performed according to the technique previously described by Pinosky et al. [14]. The following nerves will be blocked bilaterally: the supraorbital and supratrochlear nerves will be blocked with 2 ml of solution injected, at the point where they emerge above the orbit, above the midline of the eyebrow perpendicular to the skin and superficial to the periosteum; the zygomaticotemporal nerves will be blocked using 2 ml of solution midway between the supraorbital and auriculotemporal nerves; the auriculotemporal nerves will be blocked with 3 ml of solution of 1.5 cm in front of the ear at the level of the tragus. The needle will be perpendicular to the skin and 1.5 ml will be injected in the deep fascia, while another 1.5 ml will be superficially injected during the needle extraction; the postauricular branches of the greater auricular nerves will be blocked with 2 ml of solution 1.5 cm posterior to the tragus of the ear, between the skin and bone; the greater, lesser, and third occipital nerves will be blocked with 2 ml of solution injected along the superior nuchal line, approximately midway between the occipital protuberance and the mastoid process. The total volume of the solution used for scalp nerve blocks will be 22 ml in all participants.

### Criteria for discontinuing or modifying allocated interventions {11b}

Prior to providing consent, it will be explained that participation in this clinical trial is voluntary and that each participant has the right to withdraw from the study at any time, without stating reasons, without incurring disadvantages for their future care or benefits. Participants can withdraw from the study at their own request or at the principal investigator’s (PI) discretion. Withdrawal of consent can result in the termination of patient’s participation. No further study-related measures will be carried out. The PI will be responsible for any amendment of the trial. Any protocol amendment will be submitted by PI and approved by the Institutional Review Board (IRB).

### Strategies to improve adherence to interventions {11c}

During preoperative visit, patients will be explained on how to use the PCA pump. The PI will supervise the implementation of the intervention and follow-up throughout. A blinded research assistant will conduct postoperative visits at 2, 4, 8, 12, 16, 20, 24, and 48 h for postoperative data collection and ensure adherence to interventions. The blinded postoperative care staff will also regularly check on participants and will advise them to press the PCA demand button if necessary. To monitor and improve adherence, there will be online updates of PCA drug dosage, press counts, and time of each press at real time.

### Relevant concomitant care permitted or prohibited during the trial {11d}

On arrival to the postanesthesia care unit (PACU) or intensive care unit (ICU), each patient will receive patient-controlled analgesia (PCA) pumps including 100 μg sufentanil and 16 mg ondansetron diluted to 100 mL of 0.9% saline. Postoperatively, when the patient reports a numeric rating scale (NRS) score of 4 or more, or at the request of the patient, patients will be treated with PCA 2 μg bolus, with a lockout interval of 10 min, without continuous background infusion dose or loading dose, and a maximum of 8 μg per hour. The type and doses of postoperative analgesic supplementation will be recorded in detail in the case report form (CRF).

### Provisions for post-trial care {30}

All types of AEs that occur from the day of randomization to the end of follow-up will be reported to the sponsor as soon as possible after the research staff identifies the event. Meanwhile, patients will also be treated as soon as possible if any AEs occur during the trial. The drug combinations, such as the drug name, frequency of use, dosage form, and dose, will be recorded in detail by researchers.

### Outcomes {12}

#### Primary outcome

The primary outcome will be the duration of analgesia, defined as the time between the performance of the block and the administration of the first press of PCA demand button postoperatively.

#### Secondary outcomes

- The cumulative amount of sufentanil consumption by PCA at 4, 12, 24, and 48 h postoperatively and the number of participants who have no PCA press will also be documented.
- The NRS (11-point scale in which 0 = no pain to 10 = worst imaginable pain) will be determined at fixed intervals after the procedure, i.e., 2, 4, 8, 12, 16, 20, 24, and 48 h. An NRS score ≥4 will be considered significant or moderate pain. An NRS score ≥7 will be considered severe pain. Meanwhile, the localization of the site of the pain will also be documented.
- The GCS [40] will be assessed at predetermined intervals after the procedure, i.e., 2, 4, 8, 12, 16, 20, 24, and 48 h.
- Postoperative observation: postoperative nausea and vomiting (PONV), bradycardia, hypotension, and emergence delirium within 48 h after surgery will be recorded. Vomiting will be defined as the forceful expulsion of gastric contents, and nausea will be defined as an unpleasant sensation associated with the urge to vomit. Bradycardia will be defined as HR< 60 beats/min in at least two instances more than 5 min apart. Hypotension will be defined as any of the following: systolic BP < 90 mm Hg for 5 min or a 35% decrease in mean arterial blood pressure [41]. Emergence delirium will be assessed by the Sedation Agitation Scale (SAS), a 7-point scale on which a higher score represents greater agitation [42]. The time in the PACU and the length of stay (LOS) will be recorded. The LOS will be defined as the number of nights spent in the hospital after surgery. The postoperative data will be collected by reviewing each patient’s medical record.
- Postoperative satisfaction: Patient satisfaction will be assessed by the patient satisfaction score (PSS) (0 for unsatisfactory to 10 for very satisfactory) at 2, 4, 8, 12, 16, 20, 24, and 48 h after surgery.

### Participant timeline {13}

The participant timeline is shown in Table 1.

**Table 1:** Study visits of the REDUCE trial. *NRS*, Numeric Rating Scale; *PSS*: patient satisfaction score; *GCS*, Glasgow Coma Scale score; *LOS*, length of stay; *PACU*, postoperative care unit; *PONV*, postoperative nausea and vomiting; *HR*, heart rate; *MAP*, mean arterial pressure; *AEs*, adverse events; *SAEs*, serious adverse events

	Study period											
	Enrollment	Allocation	Post allocation									
Time point	Preoperative	0 d	surgery	2 h	4 h	8 h	12 h	16 h	20 h	1d	2d	Discharge
Enrollment												
Eligibility screen	X											
Informed consent	X											
Allocation		X										
Interventions												
Scalp nerve blocks with bupivacaine		X										
Scalp nerve blocks with bupivacaine plus 4 mg dexamethasone		X										
Assessments												
Baseline variables	X	X	X									
Intraoperative data			X									
The duration of analgesia										X	X	
NRS score				X	X	X	X	X	X	X	X	
GCS score				X	X	X	X	X	X	X	X	
PSS				X	X	X	X	X	X	X	X	
LOS												X
PONV				X	X	X	X	X	X	X	X	
Bradycardia				X	X	X	X	X	X	X	X	
Hypotension				X	X	X	X	X	X	X	X	
Emergence delirium				X	X	X	X	X	X	X	X	
HR and MAP			X									
Occurrence of AEs and SAEs			X	X	X	X	X	X	X	X	X	X

### Sample size {14}

Based on previous studies [19–21, 43] and our clinical practice, the average duration of analgesia after supratentorial craniotomy was approximately 600 min with a standard deviation (SD) of 240 min for patients who received scalp nerve blocks with a local anesthetic alone (the control group). A difference of 60 min in average duration of analgesia is considered the minimally clinically significant difference. Sample size calculation will be performed based on the hypothesis that the duration of analgesia in the patients receiving scalp nerve blocks with local anesthetics and 4 mg dexamethasone (the DEX_4mg_ group) will be a least 30% longer than that in the control group. Based on this assumption, we will need 70 patients per group for a power of 90% and an *α* error of 0.05; to compensate for an attrition dropout rate of 10%, 78 patients will be needed in each group and the total sample size will be 156 patients in the REDUCE trial.

### Recruitment {15}

If the team providing care considers the patient eligible for this study, they can refer the patient to the study researcher. Informed consent will be required from all patients before inclusion in the study and each participant will be informed that their participation would be voluntary and they will be able to withdraw from the study at any time. Patients will be screened and randomly assigned to a study group once scheduled for elective craniotomy under general anesthesia.

## Assignment of interventions: allocation

### Sequence generation {16a}

SPSS software version 25 will be used to carry out sample randomization with a 1:1 ratio to one of the two groups by a statistical assistant, who will not be involved in patient assessments.

### Concealment mechanism {16b}

To maintain blinding, an opaque, sealed, sequentially numbered envelope will be assigned to each patient and will be generated and opened by an independent research fellow who is not involved in patient evaluations.

### Implementation {16c}

After obtaining written informed consent and before surgery, a random envelope will be opened by an independent research investigator who will not be involved in any stage of this trial. Eligible participants will be assigned to either the control group or the DEX_4mg_ group. The random allocation number assigned will be recorded in an electronic chart.

## Assignment of interventions: blinding

### Who will be blinded {17a}

Participants, surgeons, and nurses will be blinded to the treatment assignment, along with the outcome assessors and data analysts. A clinical resident who is not involved with group allocation will be responsible for follow-up assessments as a part of daily clinical practice.

### Procedure for unblinding if needed {17b}

The envelope will be kept separately from the patient’s CRF and opened when the doctors need information about the patient’s treatment in any potentially harmful situation.

## Data collection and management

### Plans for assessment and collection of outcomes {18a}

After obtaining written informed consent, baseline data, including age, sex, BMI, intraoperative opioid dose, operation time, anesthesia time, surgical site, tumor types, and tumor size, will be collected by an independent researcher. The PCA press will be recorded by an electronic memory system in real time. The information of PCA press including the first press of PCA demand button and the cumulative amount of sufentanil consumption can be directly extracted. In order to ensure postoperative analgesia quality, an acute pain management team will monitor the pain pump remotely and deal with problems, such as congestion and battery drain, on time. An independent clinical resident who is not involved with the group allocation will complete a follow-up at 2, 4, 8, 12, 16, 20, 24, and 48 h after surgery.

### Plans to promote participant retention and complete follow-up {18b}

The patients will receive extensive information about study procedures and follow-up plans. Once a patient is enrolled or randomized, the researcher will make all reasonable efforts to follow the patient throughout the study period.

However, patients are still allowed to stop at any time during the study and are not obliged to give a reason to discontinue.

### Data management {19}

All information specified by the protocol will be collected and recorded on the CRF during the trial by the investigators or by designated representatives. The completed CRF will be reviewed and signed by the investigator or by a designated subinvestigator. An explanation will be provided for any missing data. An annual summary of the progress of the trial will be submitted to the accredited IRB.

### Confidentiality {27}

An anonymous dataset will be created by encoding each subject’s identity with a digital code. Individual participant data that underlie the results reported in this article, after de-identification (text, tables, figures, and appendices) will be shared.

### Plans for collection, laboratory evaluation, and storage of biological specimens for genetic or molecular analysis in this trial/future use {33}

This trial does not involve collecting, laboratory evaluation and storage of biological specimens for genetic or molecular analysis.

## Statistical methods

### Statistical methods for primary and secondary outcomes {20a}

Normality and homogeneity of the data distribution will be evaluated using the Shapiro Wilkinson test. Baseline characteristics, when they consist of descriptive variables with a normal distribution, will be described using the mean ± SD. Data with a skewed distribution will be presented as the median with the 25th and 75th percentiles. Dichotomous and categorical data will be described as frequencies and percentages. Group comparisons will be performed by using Student’s *t* test or the nonparametric Wilcoxon-Mann-Whitney test for quantitative variables and Pearson’s chi-squared test or Fisher’s exact test for categorical variables. NRS score and GCS at different time points will be analyzed with repeated measures analysis of variance (ANOVA) using time as the between-subjects factor. Details of statistical analysis will be fixed, at the latest, in the statistical analysis plan that will be prepared before the database is locked and analysis is commenced. The statistical tests will be 2-tailed and *P* < 0.05 will be considered statistically significant. SPSS software (version 25) will be used to conduct all analyses.

### Interim analyses {21b}

Although there are no anticipated problems that may be detrimental to the participants, serious life-threatening AEs leading to prolonged hospital stay or death will be reported to the IRB and our study will be terminated immediately.

### Methods for additional analyses (e.g., subgroup analyses) {20b}

For the primary outcome, multivariate analysis will be used to determine possible confounding factors, such as age, sex, BMI, intraoperative opioid dose, operation time, anesthesia time, surgical site, tumor types, and tumor size. Subgroup analysis will be conducted to evaluate outcomes in patients based on the site of surgery (age, sex, duration of surgery, preoperative pain severity).

### Methods in analysis to handle protocol non-adherence and any statistical methods to handle missing data {20c}

We will perform a modified intention-to-treat (ITT) analysis and participants who are randomized after enrollment and receive at least one of the study interventions will be analyzed. For per-protocol (PP) analysis, participants who withdraw from the study will be excluded from the analysis. Sensitivity analysis will be performed as an additional evaluation with the PP set, and the results will be compared with those of the modified ITT analysis. Missing observations will be replaced with the last observation carried forward.

### Plans to give access to the full protocol, participant level-data and statistical code {31c}

Study protocol, statistical analysis plan, analytic code will be available beginning 9 months and ending 36 months after article publication. Investigators whose proposed use of the data has been approved by an independent review committee identified for this purpose. Proposals should be directed to 13611326978@163.com. Meanwhile, to gain access, data requestors will need to sign a data access agreement.

## Oversight and monitoring

### Composition of the coordinating center and trial steering committee {5d}

This is a single-center study designed, performed, and coordinated in the Beijing Tiantan Hospital. Day-to-day support for the trial will be provided by a multi-disciplinary research team. PI (FL) will supervise all aspects of the study. Data managers (research coordinators) will capture, monitor, and safeguard the trial data. Acute pain management team will be routinely informed of any adverse events reported by patients or nurses and, if needed, will contact and follow-up with participants. The study team will meet biweekly.

The Trial Steering Committee (TSC) will include the PI, an independent chair, an independent clinician, and an independent statistician. The TSC will meet at least once a quarter to monitor the trial processes. The committee will check compliance with the assessment and training protocols and schedules and will oversee and manage the trial. TSC will verify trial processes, such as participant enrollment, informed consent, eligibility, assignment of participants to groups, and adherence to trial interventions.

### Composition of the data monitoring committee, its role and reporting structure {21a}

The Data Monitoring Committee (DMC) will comprise of a statistician, a neurosurgeon, an anesthesiologist, and a pain physician. They will conduct an independent review of data collection and patient safety. The DMC will report directly to TSC at their meeting after every 25%, 50%, 75%, and 100% of patient inclusions.

### Adverse event reporting and harms {22}

An AE will be defined as any untoward medical occurrence such as local hematoma, nerve injury, intra-arterial injection, allergic or toxic reaction, or facial nerve paralysis from scalp nerve blocks. Patients with blurry vision and tinnitus will be carefully observed postoperatively. Meanwhile, any signs of glucocorticoid-associated systemic toxicity such as infection will also be addressed according to the current practices and will be recorded. SAEs will include death, immediate life-threatening conditions, coma, inpatient hospitalization, or prolongation of the existing hospitalization. Serious adverse events (SAEs) occurring in the REDUCE trial must be signed and submitted to the trial management committee within 24 h after they are recognized. All SAEs that occur (including SAEs in withdrawn participants) must be continuously monitored until there are no signs or symptoms or until the participants are in stable condition. Anonymized data on all AEs must be annually reported to the IRB.

### Frequency and plans for auditing trial conduct {23}

The IRB will monitor the research process of the REDUCE trial regularly and the progress report will be submitted to IRB annually.

### Plans for communicating important protocol amendments to relevant parties (e.g., trial participants, ethical committees) {25}

Any amendments will be submitted to the IRB, and the IRB will also be informed of the end of the REDUCE trial. All changes will be recorded. Any change will be applied to all subsequent patients, and the registration record will be updated.

### Dissemination plans {31a}

At the completion of the trial and following publication of the primary and secondary outcomes, requests for data sharing will be considered by the REDUCE trial Management Group. The results of the trial will be published in peer-reviewed journals. Both positive and negative results will be reported.

## Discussion

This randomized controlled trial will evaluate the effects of dexamethasone as an additive to bupivacaine in scalp nerve blocks for postcraniotomy pain relief. The REDUCE trial will employ scalp nerve blocks by blocking several different nerves on both sides and describe a postcraniotomy analgesia method for scalp nerve blocks with improved quality and prolonged duration.

Regarding the “off-label” use of perineural adjuvants, the safety of dexamethasone is rather promising. So far, no clinical trial has reported neurotoxicity due to perineural dexamethasone. In the in vivo models, dexamethasone could produce reversible nerve block without long-term motor or sensory deficits or sciatic/dorsal root ganglion damage after a single injection or even continuous injection for 15 days [44]. More reassuringly, epidural injection of glucocorticoid is a common method for the treatment of cervical and lumbar nerve root pain and the safety of adding dexamethasone to epidural bupivacaine has already been proved [45]. The neurological risk of local dexamethasone, if any, seems small. This study will also undoubtedly pay close attention to the AEs of perineural dexamethasone.

The current protocol also has some limitations. First, we will use a single dose of dexamethasone and will not clarify the dose-effect relationship between the dose of dexamethasone and the effectiveness of pain relief. Second, we do not measure the blood concentration of dexamethasone. Systemic administration of dexamethasone has often been used for reducing cerebral edema and as a prophylaxis for PONV in clinical practice. A single dose of dexamethasone is also unlikely to cause systemic toxicity. According to previous literature, only 4 mg of dexamethasone will be administered. Third, all drugs that are part of the scalp block will not be administered on the basis of the patient’s weight. Although BMI will be considered in the multivariate analysis, it is still an inevitable potential confounding factor. Varin et al. reported that the distribution of ropivacaine after a femoral nerve block was significantly affected by body weight, but none of the pharmacodynamic model parameters showed weight dependence [46]. Whether the distribution of local anesthetics after scalp nerve blocks is significantly affected by body weight is yet to be investigated. Finally, this is a single-center trial, and it will be necessary to carry out a multicenter clinical study to provide higher levels of evidence.

If the results are positive, we may validate the use of dexamethasone as an effective adjuvant to local anesthetics for alleviating postoperative craniotomy pain and prolonging the duration of nerve blocks without associated side effects. The results of the REDUCE trial may influence future guidelines on postoperative analgesic techniques for craniotomy.

## Trial status

The research protocol (protocol version 3.2/2018-07-05) is approved by the IRB of Beijing Tiantan Hospital, Capital Medical University. Recruitment of patients for the REDUCE trial started in December 2020, and the trial is expected to be complete in 2022.

## Supplementary Information

**Additional file 1.**

## Abbreviations

NSAIDs: Nonsteroidal anti-inflammatory drugs
SPIRIT: Standard Protocol Items: Recommendations for Interventional Trials
ASA: American Society of Anesthesiologists physical status
GCS: Glasgow Coma Scale
AEs: Adverse events
HR: Heart rate
BP: Blood pressure
MAP: Mean blood pressure
IRB: Institutional Review Board
PACU: Postanesthesia care unit
ICU: Intensive care unit
PCA: Patient-controlled analgesia
NRS: Numeric Rating Scale
CRF: Case report form
PONV: Postoperative nausea and vomiting
SAS: Sedation Agitation Scale
LOS: Length of stay
PSS: Patient satisfaction score
SD: Standard deviation
ANOVA: Analysis of variance
DMC: Data Monitoring Committee
SAEs: Serious adverse events
CONSORT: Consolidated Standards of Reporting Trials
