# Supplementary material for: REDUCE trial: the effects of perineural dexamethasone on scalp nerve blocks for relief of postcraniotomy pain—a study protocol for a randomized controlled trial
Source: Trials. 2021 Nov 4;22:772. doi: 10.1186/s13063-021-05747-y (PMC8567555; doi:10.1186/s13063-021-05747-y)
Supplement: Supplementary file 1 — Additional file 1. [file 13063_2021_5747_MOESM1_ESM.docx]

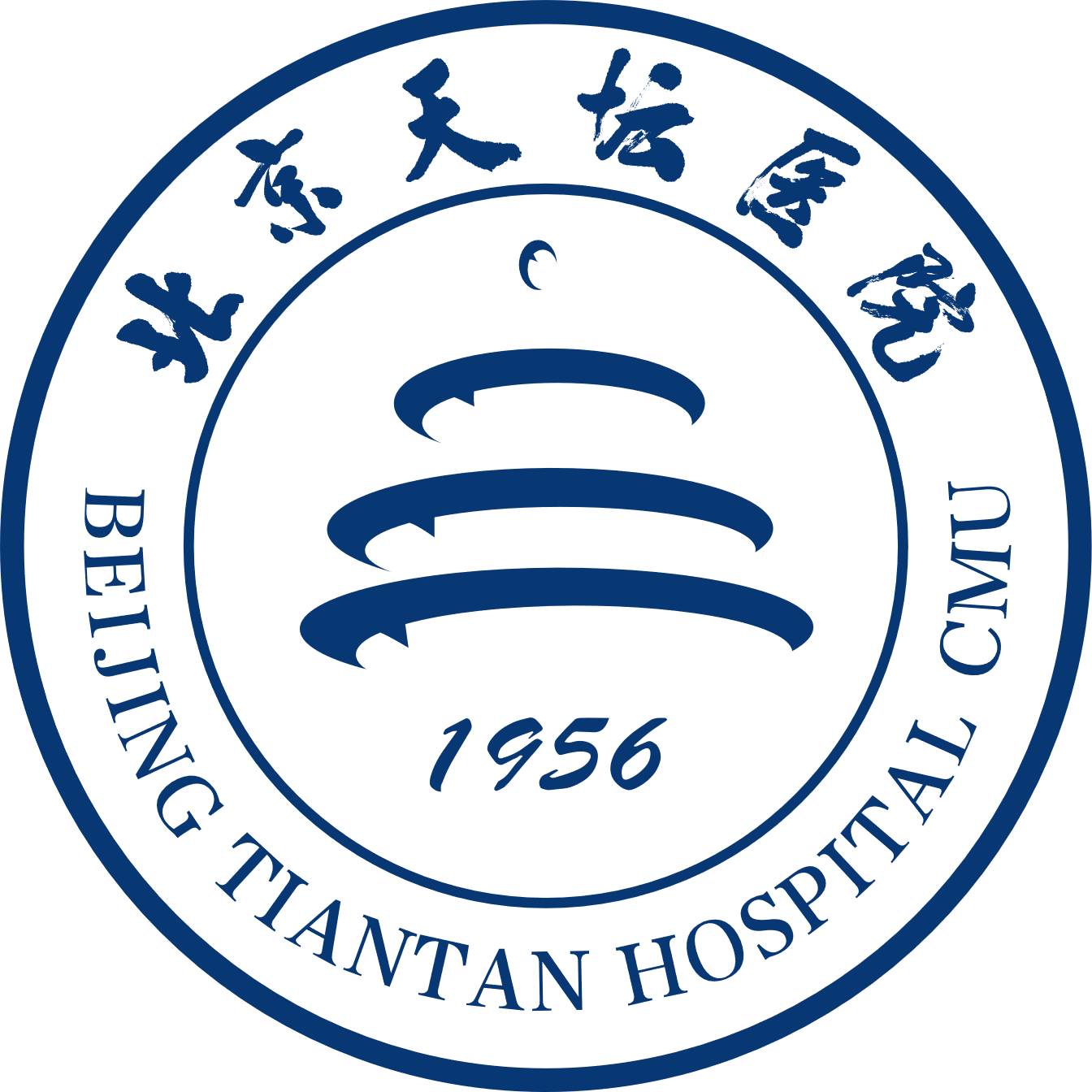


**INFORMED CONSENT FORM**

**Study title****: REDUCE trial**: The Effects of Perineural Dexamethasone on Scalp Nerve Blocks for Relief of Postcraniotomy Pain: a study protocol for a randomized controlled trial

**Organization：**Beijing Tiantan Hospital, Capital Medical University

**CRO：**Fang Luo, M.D.

**Version：**V1.1

**Date：**2020.12.01

**[Informed Consent Form for REDUCE trial:** The Effects of Pe**r**in**e**ural **D**examethasone on Scalp Nerve Blocks for Relief of Postcraniotomy Pain: a st**u**dy proto**c**ol for a randomiz**e**d controlled trial

**This Informed Consent Form has two parts:**

- **Information Sheet (to share information about the study with you)**
- **Certificate of Consent (for signatures if you agree that you may participate)**

**You will be given a copy of the full Informed Consent Form**

**PART I: Information Sheet**

**Introduction**

Dear patient~~s~~:

I am a doctor conducting a research regarding "**REDUCE trial:** The Effects of Pe**r**in**e**ural **D**examethasone on Scalp Nerve Blocks for Relief of Postcraniotomy Pain: a st**u**dy proto**c**ol for a randomiz**e**d controlled trial". I am going to give you information and request you to participate in this REDUCE trial. The REDUCE trial will recruit participants from Beijing tiantan hospital in China, and it is estimated that 98 patients will voluntarily participate in this study. The REDUCE trial has been approved by the Institutional Review Board of Beijing Tiantan Hospital Affiliated to Capital Medical University.

You do not have to decide right away, whether or not you should participate in the research. You can talk to anyone you feel comfortable with, and collect all the necessary information regarding this research before giving us your consent.

There may be some words that you may not understand. Please do not hesitate to ask me to stop at any point, as we go through the information and I will try my best to answer all of your questions. If you have any further questions, you can ask me, the study doctor or the staff.

1. **Study purpose**

Pain after neurosurgical procedures is more severe than expected. Opioids are commonly used for postoperative analgesia, but the use of opioids for neurosurgical procedures is limited by potential side effects such as sedation, miosis, nausea, vomiting and respiratory depression. Scalp nerve blocks may enhance early postoperative recovery. However, scalp nerve blocks improve postoperative analgesia for a maximum of only six hours after craniotomy. Peripheral dexamethasone significantly prolonges the duration of analgesia of various nerve blocks (e.g., saphenous nerve block, adductor canal block, thoracic paravertebral block, brachial plexus nerve block) without any unwanted effects. The analgesic effect of single dexamethasone for scalp nerve blocks without the backdrop of perioperative glucocorticoid deserves further clarification. In order to evaluate the effectiveness and safety of this technique, a randomized controlled trial should be carried out.

**2. How many patients (subjects) are expected to take part in this study?**

We will invite 98 patients to participate in this study, and the study will be conducted in Beijing Tiantan Hospital in China

**3. How long will this research last?**

The REDUCE trial will take place over a period of 2 years.

**4. What kind of research procedures will I receive if I agree to participate in this study?**

If you decide to participate, you will receive the following tests to further confirm your eligibility for the study:

• physical examination and medical history inquiry;

• vital signs (e.g. breathing rate, body temperature, heart rate, etc.);

• electrocardiography.

If you meet the inclusion criteria, you will be randomly divided into either the control group or the DEX_4mg_ group. The participants assigned to the control arm, will receive scalp nerve blocks with 0.5% bupivacaine 21 ml with epinephrine at 1:200,000, plus normal saline 1 ml. Participants in the DEX_4mg_ arm will receive scalp nerve blocks with 0.5% bupivacaine 21 ml with epinephrine at 1:200,000, plus 4 mg dexamethasone (1 ml). Postoperative analgesic drug consumption, postoperative pain score, etc. will be recorded.

All subjects will be randomly assigned a number, and will have equal opportunity to be assigned to either of the two groups. The treatment group cannot be selected by yourself, your family or the researcher. During the study period, neither the participant nor the outcome assessors will be informed of the treatment method you will receive.

For an unblinded research, the groupings will not be known by the participants or the outcome assessors and data analysts in the study. The results will only be compared after the end of data collection.

We will collect the patients’ responses to treatment and health status throughout the course of the study. During the study period, the research assistants will monitor the participants’ completion and deliver reminders as necessary through telephone calls or social media software. The research assistants will be available 24 hours a day, 7 days a week, to answer any questions related to the study.

**5. What exactly will be done to me in this study?**

You may be treated with a standardized treatment protocol based on the current recommendation for scalp nerve blocks with local anesthetics.

**6. Do I have any other treatment options? If I want to stop participating in the study, what should I do?**

If you do not wish to participate in this study, you will be treated based on the current recommended analgesic protocol for scalp nerve blocks with local anesthetic. Additional treatments involve routine postoperative analgesics.

Participation in this study may or may not improve your recovery process.

You have the right to drop out of the study at any point throughout the study.

**7.Who can take part in this study?**

All willing participants who fulfill all of the following criteria will be participating in this study:

- Patients scheduled for elective supratentorial craniotomy under general anesthesia
- Aged 18 to 64 years;
- An American Society of Anesthesiologists (ASA) physical status of I, II or III;
- A preoperative Glasgow Coma Scale (GCS) score of 15/15.
- Be able to understand the nature and potential individual consequences of the clinical trial.

**8. Who will be** [**exclude**](javascript:;)**d to take part in this study?**

Patient with any of the following conditions will be excluded from participation:

- History of chronic headache or chronic pain syndrome of any cause, psychiatric disorders or uncontrolled epilepsy;
- Inability to understand or use the pain scales before surgery;
- Excessive alcohol or drug abuse, chronic opioid use (more than 2 weeks or 3 days per week for more than 1 month), use of drugs with confirmed or suspected sedative or analgesic effects, or use of any painkiller within 24 hours before surgery;
- Request of oral/intravenous glucocorticoid to decrease cerebral edema within 1 week before surgery;
- Pregnancy or breastfeeding;
- Extreme body mass index (BMI) (< 15 or > 35);
- Participation in another interventional trial that interferes with the intervention or outcome of this trial;
- Refusal or inability of the patient and/or legal guardian to provide informed consent;
- Coagulopathy;
- Infection around the puncture site;
- History of allergies to any of the study drugs.

**9. What side effects or discomfort will I face by taking part in the study? What will the researchers do to protect me against these risks?**

Based on our pilot study, no specific adverse events were observed for dexamethasone in combination with bupivacaine, and no drug interactions were detected. During the study period, the principal investigators will monitor all AEs and SAEs in real time. The safety precautions in our study will be further adjusted based on the study process and AE reports. In addition, treatment may be ineffective, or may lead to the development of diseases or a combination of diseases. However, we will monitor you closely with regular follow ups and keep track of any unwanted side effects or problems. We will give you a telephone number to call if you notice anything out of the ordinary, or if you have any concerns or questions. You can also come to our clinic at any time. We may use some other medication to reduce the symptoms of the side effects or reactions. If necessary, we will discuss it together with you and you will always be consulted before we move to the next step.

**10. How can I benefit from this study? How could others benefit?**

If you participate in this research, postoperative pain may be reduced. There may not be any other benefit for you but your participation is likely to help us find the answer to the research question, and help others’ recovery process later in the future.

**11. Do I need to pay for the study?**

In order to compensate for the inconvenience for you, the study will pay for the treatment/ examination expenses and registration fees during your follow-up visits.

If you need to perform treatment and examination as a result of other complications or diseases, it may not be free.

You will not be provided any incentive to take part in this research. However, you will be reimbursed with (**provide a figure if money is involved**) for your lost time and travel expense.

**12. What happens if I get hurt, become sick, or have other problems as a result of this research?**

No specific adverse events were observed for dexamethasone in combination with bupivacaine, and no drug interactions were detected in our pilot study. However, if you do suffer any research-related harm during the study, please immediately inform the study physician, who will provide appropriate treatment for you. Treatment costs and economic compensation according to relevant national regulations will be provided to you by our research team.

We intend to perform regular follow-ups to observe the possible side effects/adverse reactions caused by drug treatment. While the possibility of this happening is very low, you should still be aware of them. If something unexpected happens and harm does occur, we will take measures to prevent and treat them.

Even if you have signed this informed consent, you still retain all your legal rights.

**13. What information about me could be seen by the researchers or by other people? Who might see it?**

Your medical records will be kept in the hospital, and researchers, research authorities, and ethics committees will be allowed to access your medical records. Any public report on the results of this study will not disclose your personal identity. We will, to the extent permitted by law, make every effort to protect the privacy of your personal medical data.

**14. Do I have to take part in the study? Do I have the right to refuse or withdraw?**

The decision to participate in this study is entirely voluntary. It is your choice whether you participate or not. If you choose not to consent, all the services you receive at this clinic will continue and nothing will change. You may also choose to change your mind and stop participating at any point, even if you agreed earlier, and the services you receive at the clinic will continue and you could receive alternative therapies, such as postoperative analgesic.

If you decide to withdraw from this study, please contact your doctor in advance. In order to ensure your safety, you may be required to carry out some related tests, which is beneficial for your health.

**15. How will participating in this study affect my life?**

Some scheduled follow-up visits may be inconvenient for you, we will try our best to avoid such inconvenience. In addition, some tests may make you feel uncomfortable. If you have any questions about the tests or procedures, consult the study physician at any time.

Your study physician will tell you what medications you may or may not take during the study. Consult with your study physician before taking any new prescribed medications.

You are not allowed to participate in any other clinical studies involving drugs or medical devices throughout the entire duration of this study.

**16. Who to contact**

If you have any questions, you may ask the researchers at any point throughout the study. If you wish to ask questions later, you may contact [Chunmei Zhao, No.119 South 4th Ring West Road, Fengtai District, Beijing, China /59976664].

This proposal has been reviewed and approved by Beijing Tiantan Hospital Affiliated to Capital Medical University of the IRB, which is a committee tasked to make sure that research participants are protected from harm. If you wish to find out more about the IRB, contact 010-59978555.

**PART II: Certificate of Consent**

**Statement by the patient:**

**I have read the foregoing information. I have had the opportunity to ask benefits or risks about the procedure and any questions that I have asked have been answered to my satisfaction. I consent voluntarily to participate as a participant in this study.**

**I agree**□ **or disagree**□ **with the use of my medical records for any other study except this study.**

**Signature of Patient: ___________________**

**Print Name of Participant: __________________**

**Contact: __________________**

**Date: ___________________________**

**Day/month/year**

**Statement by the researcher/person taking consent**

**I have accurately read out the information sheet to the potential participant, and all the benefits or risks have been answered correctly and to the best of my ability. I confirm that the individual has not been coerced into giving consent, and the consent has been given freely and voluntarily.**

**A copy of this ICF has been provided to the participant.**

**Signature of Researcher /person taking the consent__________________________**

**Print Name of Researcher****/person taking the consent________________________**

**Contact: __________________**

**Date ___________________________**

**Day/month/year**
